# Rapid Pterygium Progression in a Child on Growth Hormone Therapy: A Case Report and Literature Review

**DOI:** 10.7759/cureus.100873

**Published:** 2026-01-05

**Authors:** Ming Chen, Shay Nakahira, Justin H Wong, Mindy Mandich

**Affiliations:** 1 Department of Ophthalmology, University of Hawaii John A. Burns School of Medicine, Honolulu, USA; 2 Department of Medicine, University of Hawaii John A. Burns School of Medicine, Honolulu, USA

**Keywords:** fibrovascular proliferation, growth hormone therapy, ocular surface disease, pediatric pterygium, ultraviolet exposure

## Abstract

Pterygium is rare in children, and the effects of growth hormone (GH) therapy on its progression have not been previously described. Because GH and GH-releasing hormone receptor pathways influence cellular proliferation and inflammation, GH supplementation may contribute to accelerated pterygium growth in susceptible patients. This case highlights a rapidly progressive pterygium in an adolescent receiving GH therapy. A 13-year-old male presented with blurred vision and a rapidly enlarging nasal pterygium in the left eye. A small red nodule had first been noted in this patient at six years of age. He received a six-month course of GH in China for short stature and later resumed GH therapy in Hawaii with a one-year prescription. During this second course, the lesion grew significantly, accompanied by worsening visual impairment. On presentation, visual acuity was measured at 20/20 OD and 20/100 OS. Slit-lamp examination showed a thick fibrovascular pterygium extending toward the pupil. The patient underwent pterygium excision with mitomycin C and amniotic membrane grafting. Four days postoperatively, pinhole visual acuity improved to 20/40 OS, with residual central corneal opacity and persistent with-the-rule astigmatism. The temporal association between GH therapy and rapid pterygium enlargement in this patient suggests a potential link between GH exposure and accelerated fibrovascular proliferation. Given the established presence of GH and GH receptors in ocular tissues, clinicians should consider GH therapy as a possible contributing factor when evaluating pediatric patients with progressive ocular surface lesions such as pterygium. Further research is needed to clarify the role of endocrine signaling in pterygium pathogenesis.

## Introduction

Pterygium is a common ocular surface disorder characterized by fibrovascular proliferation and extension of conjunctival tissue onto the cornea. It primarily affects adults, most often in the fourth and fifth decades of life, and is strongly associated with chronic exposure to ultraviolet (UV) radiation. Its pathogenesis is multifactorial, involving environmental, genetic, and molecular influences that drive epithelial proliferation, angiogenesis, and extracellular matrix remodeling [1-3].

Pterygium is rarely observed in children or adolescents, and rapidly progressive lesions in these younger patients are especially uncommon. When pterygium develops early in life, it often suggests additional systemic, hormonal, or genetic factors beyond UV exposure [4]. Familial clustering of pterygium has been documented, supporting a genetic predisposition in some individuals [5-7].

Growth hormone (GH), produced by the anterior pituitary, plays a key role in regulating growth, metabolism, and tissue repair through both direct receptor-mediated pathways and indirect stimulation of insulin-like growth factor-1 (IGF-1). GH and its receptor are expressed in ocular tissues, including the cornea and conjunctiva, where they contribute to epithelial proliferation and wound healing [8,9]. These biological effects raise the possibility that exogenous GH therapy may influence proliferative ocular surface disease processes such as pterygium. However, to date, no studies have reported an association between GH therapy and pterygium development or progression.

Although UV exposure remains the most established risk factor for pterygium, emerging evidence indicates that growth factor- and hormone-mediated signaling pathways may also contribute to its pathogenesis [4,10]. The use of GH therapy has risen more than threefold over the past two decades, most commonly for the treatment of short stature [11], underscoring the importance of understanding potential ocular effects of exogenous GH.

This case describes a 13-year-old boy who developed rapid enlargement of a pterygium following initiation of GH therapy. This report highlights a potential link between GH exposure and accelerated fibrovascular proliferation on the ocular surface and emphasizes the need for further investigation into hormonal contributions to pterygium pathophysiology.

## Case presentation

**Figure 1:**
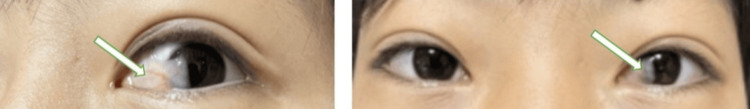
Patient’s initial presentation of pterygium along the nasal limbus of the left eye, encroaching the pupil (white arrow). Written informed consent was obtained from the patient for inclusion in this case report.

A 13-year-old otherwise healthy Chinese male presented with three months of progressively worsening blurred vision in the left eye due to a rapidly enlarging nasal pterygium (Figure 1). The lesion first appeared at age six as a small growth along the nasal limbus but had shown markedly accelerated enlargement over the year preceding presentation. The patient had received a six-month course of an unknown GH therapy in China at the age of seven years for short stature. GH therapy was subsequently restarted after relocating to Hawaii at age 12, consisting of daily subcutaneous somatropin injections (1.2 mg/day) for approximately 10 months, during which time the pterygium exhibited significant acceleration in growth. Although the pterygium was present before the initiation of GH therapy, it had not encroached upon the visual axis before treatment. The patient also reported increased sun exposure between the ages of 11 and 13.

Symptoms included left eye irritation, difficulty reading, and progressive visual impairment that interfered with daily activities. He denied systemic symptoms. Past medical, surgical, and family histories were unremarkable. Aside from GH, he took no medications, reported no drug allergies, and denied alcohol, tobacco, or drug use.

On examination, best-corrected visual acuity was 20/100 in the left eye (OS) and 20/20 in the right eye (OD). Subjective refraction measured −1.75 + 4.50 × 97 OS and −1.25 + 0.25 × 109 OD. Intraocular pressures recorded at 11:00 AM were 19.1, 13.1, and 22.5 mmHg OS, and 14.9, 10.0, and 13.7 mmHg OD. Slit-lamp evaluation revealed a thick fibrovascular pterygium encroaching onto the pupil (Figure 1). Dilated fundus examination was normal. The patient also demonstrated significant with-the-rule astigmatism.

Given the rapid progression and visual impairment, the patient elected to undergo pterygium excision. He was counseled extensively on the high risk of recurrence due to his age and lesion characteristics. He underwent standard adult pterygium excision with local anesthesia (2% lidocaine with epinephrine), intraoperative mitomycin C, amniotic membrane graft placement, and subconjunctival dexamethasone injection. The pterygium and its feeding vessel were dissected from the limbus, hemostasis was achieved with cautery, and an amniotic membrane graft was applied. Postoperatively, tobramycin/dexamethasone and besifloxacin eye drops were initiated, and a bandage contact lens was placed for three days. The procedure was uncomplicated.

Postoperative recovery progressed appropriately. The bandage contact lens was removed, and soft contact lenses were worn for one week. The patient continued topical tobramycin/dexamethasone and besifloxacin for one month and adhered strictly to postoperative instructions, including avoidance of eye rubbing, limiting environmental exposure, and consistent medication use. Pathology confirmed benign fibrovascular tissue consistent with pterygium.

**Figure 2:**
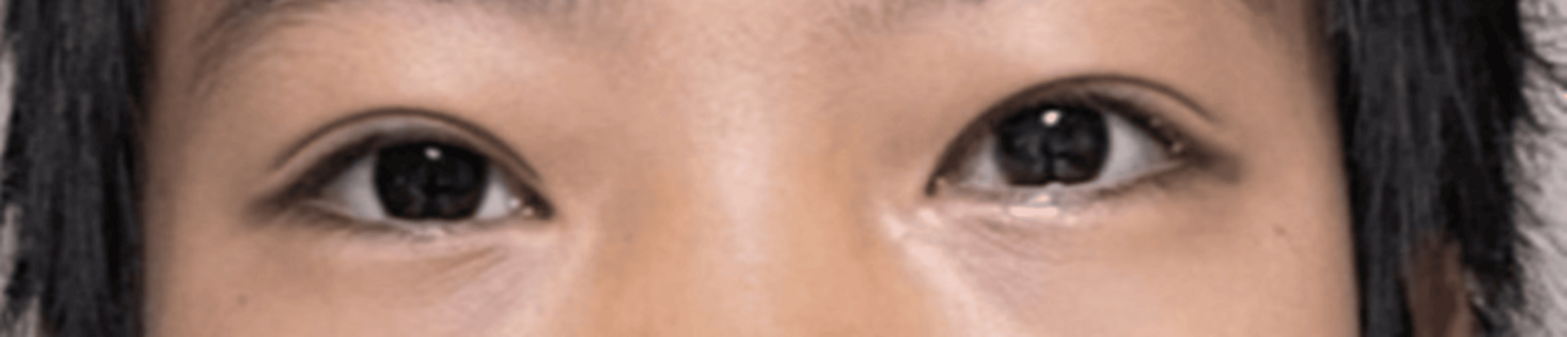
Patient at four days postoperatively with residual central corneal opacity. Written informed consent was obtained from the patient for inclusion in this case report.

At the one-week follow-up, the left eye demonstrated adequate healing with mild residual bare sclera, and the patient reported improved vision and reduced discomfort. Four days postoperatively, pinhole visual acuity improved to 20/40, with residual central corneal opacity and persistent with-the-rule astigmatism (Figure 2). Subjective refraction at that time was -7.25 + 5.75 × 59 OS.

**Figure 3:**
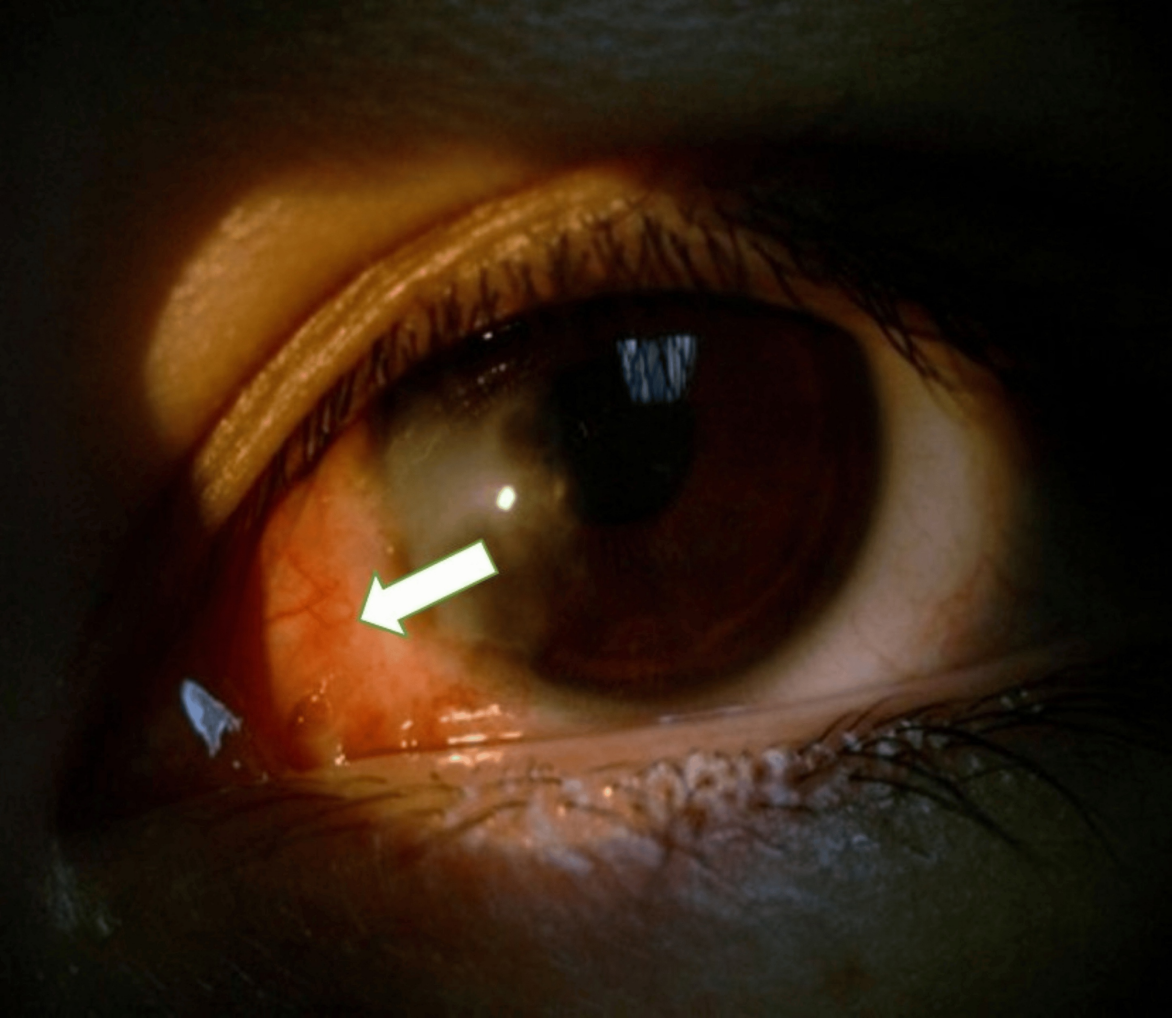
Patient at five months postoperatively with recurrent pterygium at the limbus with fibrovascular change (white arrow). Written informed consent was obtained from the patient for inclusion in this case report.

**Figure 4:**
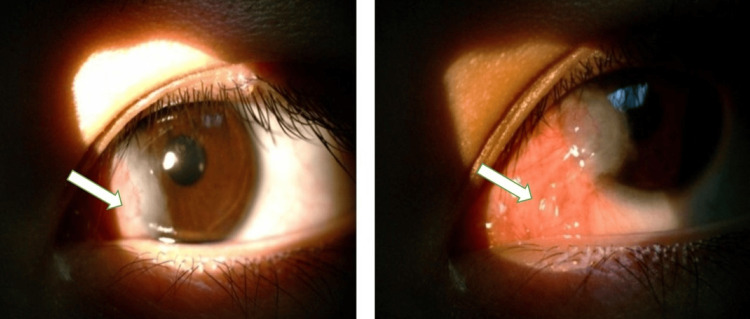
Patient at six months postoperatively presenting with significant neovascular invasion of the cornea and congested full recurrence of the pterygium (white arrow). Written informed consent was obtained from the patient for inclusion in this case report.

At the five-month follow-up, early recurrence was noted at the limbus with fibrovascular tissue formation, though no corneal neovascularization was present (Figure 3). By six months postoperatively, slit-lamp examination revealed significant corneal neovascularization with a fully recurrent, congested pterygium (Figure 4). GH therapy was discontinued six months after surgery by the patient’s endocrinologist. The patient subsequently underwent repeat surgical excision, after which no recurrence was observed at the three-month follow-up.

## Discussion

Pediatric pterygium is uncommon, and its presence typically suggests additional systemic, genetic, or environmental influences beyond UV exposure alone. The most widely accepted pathophysiologic mechanism involves UV-induced oxidative stress, which triggers chronic inflammation and upregulation of growth factors such as vascular endothelial growth factor (VEGF) and transforming growth factor-beta (TGF-β), promoting fibrovascular proliferation [1,12]. In this patient, a small unilateral conjunctival fibrovascular lesion was present as early as six years of age, raising the possibility of an underlying genetic predisposition. His relocation from China to Hawaii likely contributed to accelerated growth, given the substantial increase in ambient UV radiation. As pterygia enlarge, they may induce severe astigmatism and visual impairment, particularly when encroaching on the visual axis [13]. Surgical excision is often required, yet recurrence rates remain high, up to 36.1% within four to six months [14].

GH and IGF-1 play central roles in cellular proliferation, angiogenesis, and extracellular matrix remodeling, processes that parallel key histopathologic features of pterygium. GH acts through both direct receptor-mediated pathways and through IGF-1, activating cascades such as JAK-STAT, PI3K/Akt, and MAPK that stimulate epithelial proliferation and tissue repair [15]. Importantly, GH and its receptor are expressed in ocular tissues, including the cornea and conjunctiva [8,9], providing a biologically plausible mechanism through which exogenous GH could enhance fibrovascular growth on the ocular surface.

Experimental studies support this concept. GH and growth hormone-releasing hormone receptor (GHRH-R) signaling have been shown to increase proliferation and inhibit apoptosis in pterygium epithelial cells, whereas GHRH-R antagonists reverse these effects [16]. Additionally, GH has been shown to accelerate corneal epithelial migration and wound healing [17,18]. Taken together, these findings suggest that systemic GH therapy, particularly when combined with increased UV exposure, may synergistically potentiate angiogenic and fibroblast-mediated pathways that drive pterygium growth.

Despite this mechanistic plausibility, clinical evidence linking GH therapy to pterygium progression remains sparse. Most research focuses on VEGF, fibroblast growth factor 2, and TGF-β-related pathways, while the GH/IGF-1 axis has been far less explored [19]. Prior studies evaluating IGF-1 expression in pterygium tissue have yielded inconsistent results [4], underscoring gaps in our understanding of endocrine influences on ocular surface disease.

This case contributes to the limited clinical literature by highlighting a temporal association between exogenous GH therapy and rapid pterygium enlargement in a pediatric patient. While causation cannot be established from a single observation, the findings support the hypothesis that GH may serve as a cofactor in fibrovascular proliferation among susceptible individuals, particularly those with significant UV exposure or underlying predisposition. Further research is warranted to clarify the role of GH signaling in pterygium pathogenesis and to determine whether GH therapy may influence recurrence or progression in pediatric and adult populations.

## Conclusions

This report describes an unusual, rapidly progressive pterygium in a pediatric patient receiving GH therapy. The temporal relationship and mechanistic plausibility suggest a potential role for GH signaling in accelerating fibrovascular proliferation on the ocular surface. The known expression of GH receptors in ocular tissues and their involvement in fibroblast activation, collagen deposition, and angiogenesis provide a biologic framework supporting this association. The combined influence of GH/IGF-1 signaling and environmental stressors such as UV exposure may explain the unusually aggressive clinical course observed. Further clinical and experimental studies are warranted to clarify the relationship between GH therapy and pterygium progression, particularly in pediatric populations. Clinicians should remain aware of this potential association when monitoring patients on GH therapy who develop ocular surface changes.
